# Common pathogenic mechanisms in the hippocampus across neurodegenerative dementias: Alzheimer’s disease, Down syndrome, and Parkinson’s disease

**DOI:** 10.1038/s44400-026-00075-x

**Published:** 2026-04-29

**Authors:** René A. J. Crans, Marta Fructuoso, Karen Bascón-Cardozo, Hatice Recaioglu, Jesus Sotelo-Fonseca, Yannick Vermeiren, André Strydom, Debby Van Dam, Peter P. De Deyn, Bernardo Rodríguez-Martín, Marie-Claude Potier, Mara Dierssen

**Affiliations:** 1 Center for Genomic Regulation (CRG), The Barcelona Institute for Science and Technology, Barcelona, Spain; 2 Paris Brain Institute ICM, Salpêtrière Hospital, Paris, France; 3 Department of Biomedical Sciences, Laboratory of Neurochemistry and Behavior, Experimental Neurobiology Unit, University of Antwerp, Antwerp, Belgium; 4 Department of Forensic and Neurodevelopmental Sciences, Institute of Psychiatry, Psychology & Neuroscience, King’s College London, London, United Kingdom; 5 Department of Neurology and Alzheimer Research Center, University of Groningen and University Medical Center Groningen, Groningen, The Netherlands; 6 Department of Neurology and Memory Clinic, Middelheim General Hospital (ZNA), Antwerp, Belgium; 7 Department of Experimental and Health Sciences, University Pompeu Fabra, Barcelona, Spain; 8 Biomedical Research Networking Center for Rare Diseases (CIBERER), Barcelona, Spain; 9 Present Address: Division of Human Nutrition and Health, Chair Group Nutritional Biology, Wageningen University and Research (WUR), Wageningen, The Netherlands

**Keywords:** Biomarkers, Diseases, Neurology, Neuroscience

## Abstract

Extensive evidence suggests overlapping pathological mechanisms in the brain of individuals with Parkinson’s disease dementia, Down syndrome dementia, and Alzheimer’s disease. For these neurodegenerative dementias, we observed that the chronological age did not align with their biological age, which was determined based on hippocampal transcript levels (i.e., transcriptional age). Subsequently, we performed a transcriptomic analysis that corrected for the transcriptional age in the hippocampus of affected individuals, highlighting common underlying pathogenic mechanisms. There were 45 common differentially expressed genes (DEGs), whereas enriched functional terms were related to lysine *N*-methyltransferase activity and intermediate filament. Co-expression network analysis displayed a module that was significantly downregulated in the non-demented control group only. This module identified *EHMT2* and *LMNB2* as hub genes, which were also common DEGs. Overall, these findings uncover shared functional insights in the hippocampus, while specifically highlighting *EHMT2* and *LMNB2* as potential universal biomarkers or disease-altered targets across neurodegenerative dementias.

## Introduction

Dementia refers to a group of disorders causing a significant cognitive decline that is sufficient to interfere with daily life, including domestic, occupational, or social functioning^1^. The global prevalence is about 6% for individuals over the age of 60, where the number of people living with dementia is expected to increase from 57 to 152 million in the next 30 years^2–4^. Major risk factors to develop dementia are aging, genetics, and cardiovascular diseases^5^. Although Alzheimer’s disease (AD) has become almost synonymous with dementia, the latter can arise from multiple possible causes, such as neuropsychiatric, medical, and neurological conditions^6^. In older adults, dementia is mainly caused by neurodegenerative processes associated with various disorders^1^.

A growing collection of evidence suggests overlapping pathogenic mechanisms for dementias with AD, Down syndrome (DS), and Parkinson’s disease (PD). Over 90% of DS individuals have a lifetime risk to develop AD-like dementia and this is currently the leading cause of death in this population^7^. AD entails a universal progression to dementia, whereas around 30–60% of the PD patients develop dementia in later stages of the disease^8–10^. The surviving neurons and neuronal processes in most patients with Parkinson’s disease dementia (PDD) contain Lewy body (LB) inclusions, which are abnormal accumulation and aggregation of α-synuclein proteins. This typical neuropathological hallmark for PD is frequently found in AD patients as well^11^. On the other hand, clinically diagnosed cases of PD demonstrate brain amyloid beta (Aβ) accumulation at levels typically associated with AD^12–14^. Similarly, almost all middle-aged individuals with DS present both the neuropathological hallmarks of AD, which are Aβ-containing senile plaques and phosphorylated tau-containing neurofibrillary tangles (NFT)^15^. This is partly explained by the overexpression of the amyloid precursor protein that is located on the extra copy of the human chromosome 21, resulting in higher levels of amyloid depositions in the brain of individuals with DS than in sporadic AD^16,17^. Moreover, cases of DS presenting pathologic changes of PD and LB formations have been reported^18,19^. Motor control deficits in DS individuals with parkinsonism could be efficiently reversed with L-DOPA, which is the most effective drug for the symptomatic treatment of PD^20^. Palat and colleagues suggested that psychomotor slowing in individuals with DS may be mistakenly attributed to AD, but can be in fact a sign of parkinsonism, as PD is underestimated in DS^20^. Despite differences in etiology and clinical presentation between AD, DS, and PD, they might converge in their pathogenic mechanisms leading to dementia.

This study focuses on the hippocampus, a complex and plastic brain region embedded deep in the temporal lobe, playing a major role in learning and memory and which is highly susceptible to aging-related changes and dementia^21,22^. Longitudinal studies have shown increased rates of hippocampal atrophy in AD compared to age-matched non-demented controls, which has been accepted as a biomarker for sporadic AD^23^. Non-demented individuals with DS have significantly smaller volumes of the hippocampus, but not the amygdala. However, their hippocampal volume remains relatively constant in DS without dementia throughout the fifth decade. In contrast, the reduction of the hippocampal volume is considered as a clinical sign of dementia for individuals with DS over the age of 50 years^24–26^. In PDD patients, the hippocampus also shows increased atrophy with progression of the disease^27^. Hence, an increased knowledge of the underlying hippocampal mechanisms in dementia may lead to the design and application of diagnostic strategies or treatments.

Aging has been associated with changes in transcriptional regulation, which is known to be a complex molecular mechanism, characterized by the intricate interplay between genetic variants, transcription factors, and DNA methylation^28^. The alterations in transcript levels may not be directly associated with the chronological age (i.e., the number of years a person has been alive), but impacted by the biological age of an organ or tissue^29,30^. The biological age is a measure of the apparent age based on a certain aspect (i.e., DNA methylation or transcript levels) and is influenced through intrinsic and external factors, such as genomic aberrations, diet, and stress^29–32^. In 1978, DS has been postulated as a segmental progeroid syndrome, as individuals with DS suffer from several age-associated disorders much earlier than euploid persons^33,34^. A recent study reported that the biological age in adults with DS is increased with approximately 18.8 years compared to their chronological age-matched controls, whereas the rate of aging does not increase throughout their lifespan^35^. However, biological age based on transcript levels (i.e., transcriptional age) has not yet been inferred and implemented in any analytical pipeline of transcriptomic studies on post-mortem DS with dementia (DSD), AD, or PDD human brains.

In this study, bulk RNA-sequencing (RNA-seq) was performed on total RNA (i.e., non-mRNA enriched) from post-mortem frozen human brain material of demented individuals diagnosed with AD, DS, and PD. Our investigation focused on assessing the hippocampus due to its pivotal role and involvement in learning and emotion, and it’s importance for spatial, episodic, and long-term memory formation^36^. To our best knowledge, this is the first RNA-seq analysis that considers transcriptional age acceleration (RNAAge) in the transcriptome analysis pipeline and detects common underlying pathogenic mechanisms for dementias from three different neurodegenerative disorders, highlighting potential universal biomarkers or disease-altering targets.

## Results

### Descriptive statistics

A summarized description of the samples is shown in Table 1, including sex, APOE genotype status, age at death, Braak tau stage (AD Braak), Thal phase for amyloid beta plaques (Thal phase), CERAD neuritic plaque score (CERAD score), Braak α-synuclein stage (PD Braak), post-mortem interval (PMI), and RNA integrity number (RIN). There was a significant difference in mean age at death (years) between DSD and the other subject groups (One-way ANOVA, *p*-value = 0.002). There was no significant difference in the number of males and females (Fisher’s exact test, *p*-value = 0.603), RIN (One-way ANOVA, *p*-value = 0.341) or PMI (One-way ANOVA, *p*-value = 0.179) between the non-demented controls (Control) and individuals with diagnosed dementias (i.e., PDD, AD, and DSD). Among the cases analyzed, there were ten individuals with ε3ε3 APOE genotype (*n* = 10), one with ε2ε3 APOE genotype (*n* = 1), one with ε2ε4 APOE genotype (*n* = 1), five with ε3ε4 APOE genotype (*n* = 5), and three with ε4ε4 APOE genotype (*n* = 3). Of note, no individuals were found to be ε2 homozygotes. The presence of the ε4 allele (ε4ε4/ε4ε2/ε4ε3) was observed in all the individuals with AD (6/6 = 100%) followed by less dominantly observations in PDD (2/4 = 50%), DSD (1/5 = 20%), and Control (0/5 = 0%) groups.

**Table 1:** Summarized description of the cases used in this study for the human post-mortem hippocampal tissue

	Control	PDD	AD	DSD
**Sample size**	5	4	6	5
**Sex (M/F)**	1/4	1/3	3/3	3/2
**ApoE genotype** **ε4 –** **ε4 +**	100% 0%	50% 50%	0% 100%	80% 20%
**Age at death (years)**	80.4 ± 5.8 (73–89)	79.8 ± 8.3 (68–86)	72.5 ± 9.4 (62–90)	58.8 ± 6.4 (52–67)
**AD Braak**	I/II	I – VI	V – VI	V – VI
**Thal phase**	0–2	4	4–5	5
**CERAD score**	None	N/A	Moderate – Frequent	N/A
**PD Braak**	N/A	I–VI	N/A	N/A
**PMI (h)**	2.8 ± 1.7 (1.5–5.8)	14.1 ± 10.0 (3.3–24.0)	13.2 ± 9.3 (1.8–28.0)	11.5 ± 9.9 (3.0–28.5)
**RIN**	8.1 ± 1.1 (6.6–9.2)	7.1 ± 0.6 (6.5–7.8)	7.2 ± 1.2 (5.8–8.5)	7.9 ± 0.8 (6.5–8.7)

*Control* non-demented controls, *PDD* Parkinson’s disease with dementia, *AD* Alzheimer’s disease, *DSD* Down syndrome with dementia, *AD Braak* Braak tau stage, *PD Braak* Braak α-synuclein stage, *Thal phase* Thal phase for amyloid beta plaques, *CERAD score* CERAD neuritic plaque score, *PMI* post-mortem interval, *RIN* RNA integrity number, *M* male, *F* female, *N/A* not available.

### Transcriptional aging is accelerated in the hippocampus of Down syndrome with dementia

**Fig. 1:**
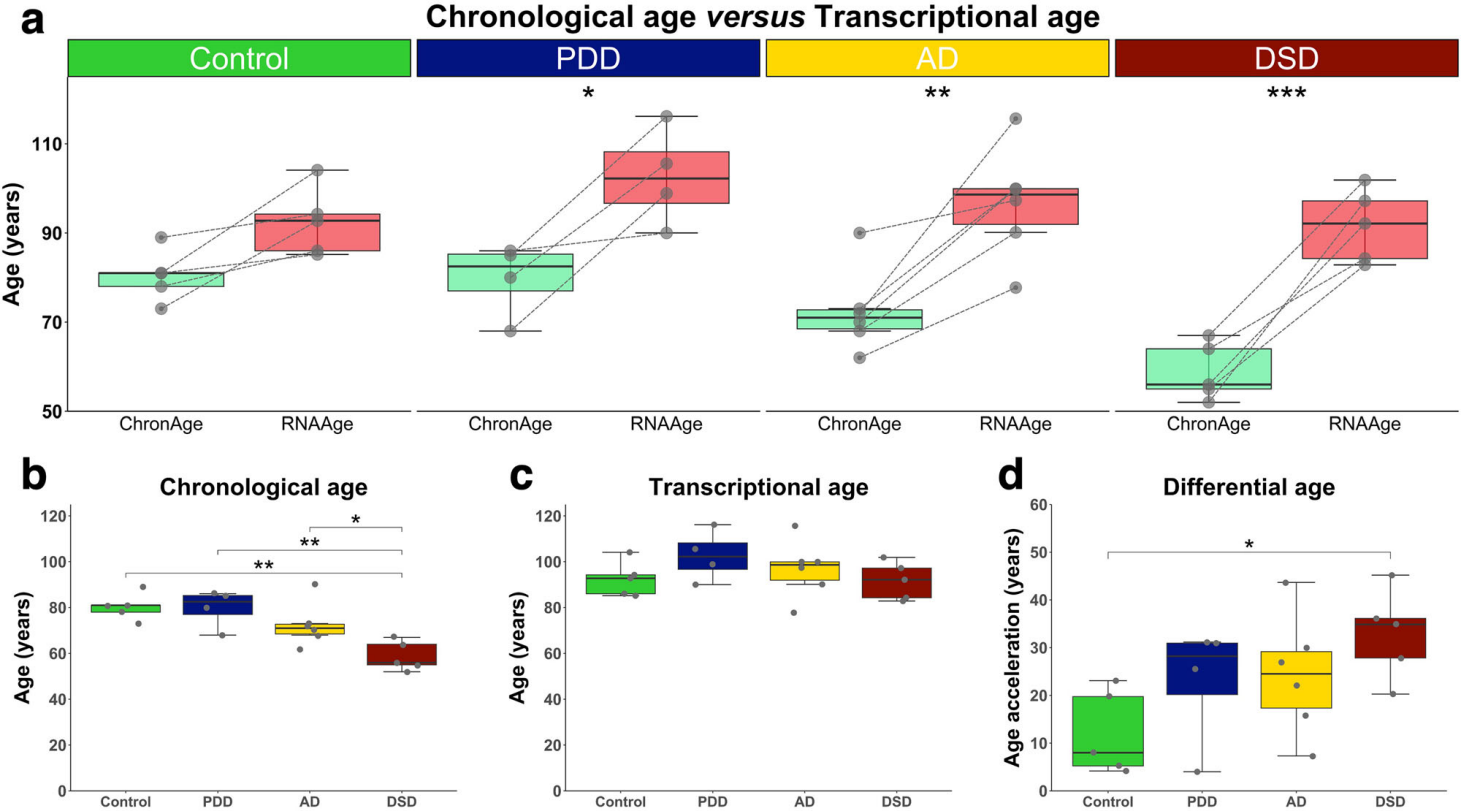
Comparisons of the chronological age, transcriptional age, and differential age. **a** Transcriptional age (RNAAge) was calculated with the RNAAgeCalc algorithm for each sample and compared against the chronological age (ChronAge) of the same individual, which are connected through a dotted line. All the dementia groups showed to have a higher transcriptional age than their biological age (Control, *p*-value = 0.427, PDD, *p*-value = 0.0211; AD, *p*-value = 0.0012; DSD, *p*-value = 0.00005). **b** Chronological age (or age at death) in years was 80.4 ± 5.8, 79.8 ± 8.3, 72.5 ± 9.4, and 58.8 ± 6.4 for the Control, PDD, AD, and DSD groups, respectively. The individuals with DS and dementia (DSD) were significantly younger at death compared to the other groups (Control *vs.* DSD, *p*-value = 0.00209; PDD *vs.* DSD, *p*-value = 0.00452; and AD *versus* DSD, *p*-value = 0.0428). **c** There was no difference in transcriptional age between the groups (92.5 ± 7.6, 102.7 ± 11.0, 96.8 ± 12.5, and 91.7 ± 8.2 for the Control, PDD, AD, and DSD groups, respectively). **d** Age acceleration was calculated by the subtraction of the chronological age from the transcriptional age, which was represented as the differential age. Individuals with DSD had a significantly acceleration in age compared to non-demented controls (Control). After normal distribution was assessed with the Shapiro-Wilk test and the assumption for homoscedasticity controlled, statistical significance was tested using the two-way ANOVA or the one-way ANOVA followed by the Tukey’s HSD *post hoc* test. Statistical significance was presented with *p-value ≤ 0.05; **p-value ≤ 0.01; ***p-value ≤ 0.001.

The transcriptional age was estimated with the RNAAgeCalc algorithm, which is a machine learning-based transcriptomic clock. This algorithm predicts tissue-specific transcriptomic age based on a fixed set of coefficients from a pre-trained elastic net model, using 1,616 age-related genes identified from a meta-analysis of the GTEx database^32^. All our hippocampal samples were of Northwest European origin (i.e., Belgium, Northern France, and England). Therefore, the model trained in brain tissue of Caucasian origin was applied for this analysis on the Control, PDD, AD, and DSD samples. Only the dementia groups showed a significantly older transcriptional age than their chronological age (Fig. 1a). The chronological age was significantly lower in the DSD group compared to the other groups (Fig. 1b). However, the transcriptional ages did not differ significantly between all groups, including controls (Fig. 1c). The mean transcriptional age in years was 92.5 ± 7.6, 102.7 ± 11.0, 96.8 ± 12.5, and 91.7 ± 8.2 for the Control, PDD, AD and DSD groups, respectively. Finally, age acceleration, as defined by the difference between biological age (e.g., transcriptional age) and chronological age (Fig. 1d), showing a significant age acceleration in DSD individuals compared to the Control group (*p*-value = 0.0386). Of note, accelerated aging in the hippocampal samples of the DSD individuals was independently confirmed with a different transcriptional age estimator, the BiT age clock algorithm (Supplementary Fig. S1)^37^. These results support that the age of individuals with DS is more appropriately represented by their biological age than by their chronological age.

### Common DEGs are linked to neurological diseases

**Fig. 2:**
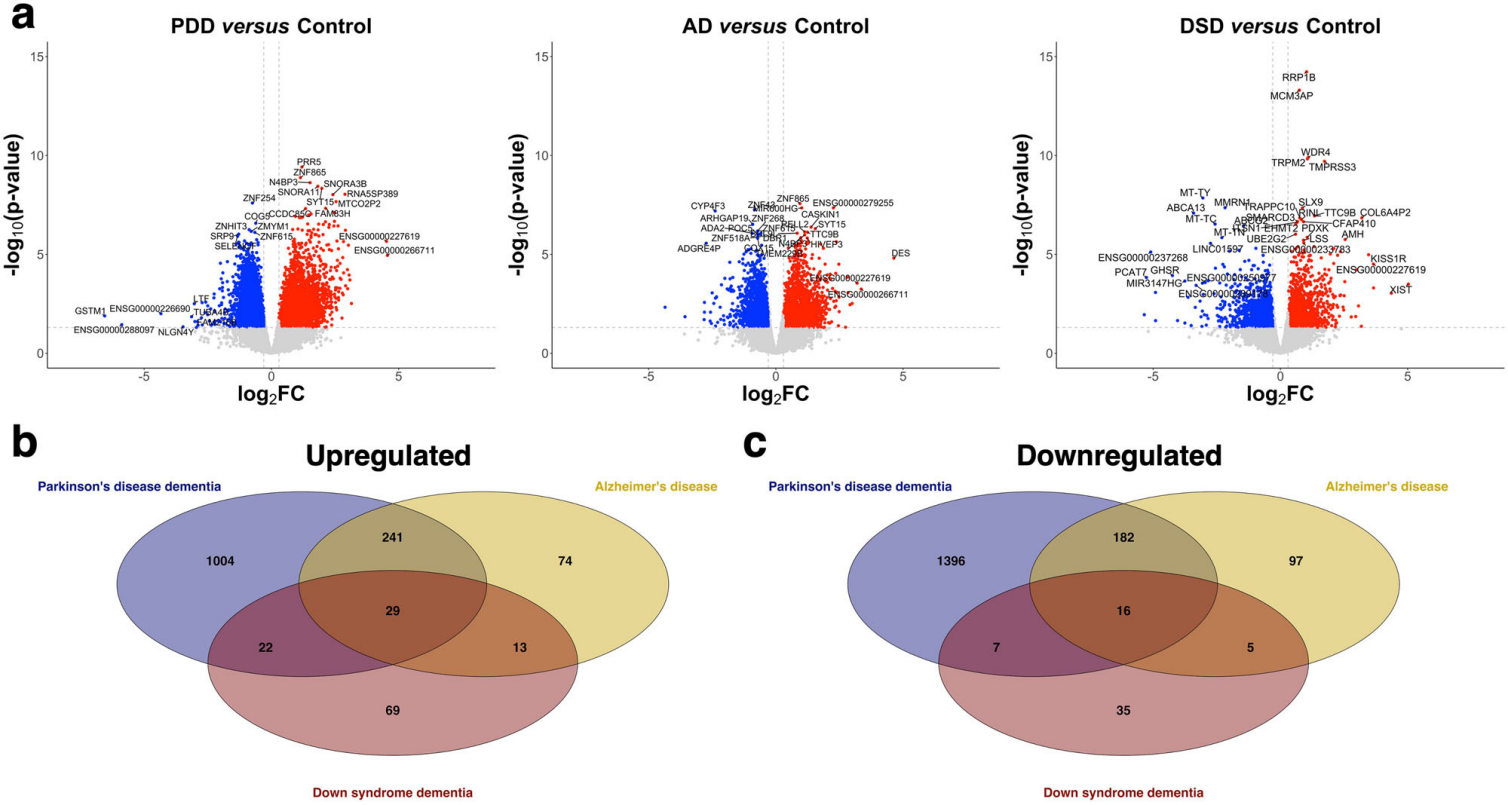
Expression analysis and overlapping differentially expressed genes for Parkinson’s disease dementia (PDD), Alzheimer’s disease (AD) and Down syndrome dementia (DSD). **a** Volcano plots showing the expression levels of differentially expressed genes in individuals with PDD, AD and DSD. Red dots indicate upregulated genes in these individuals compared to non-demented control cases (Control). In contrast, blue dots indicate downregulated genes in these individuals compared to Control. **b** Venn diagram illustrating the overlapping upregulated genes in the hippocampus of individuals with PDD, AD and DSD. In the diagram, colors represent the followings; PDD *versus* Control (blue), AD *versus* Control (yellow) and DSD *versus* Control (red). **c** Venn diagram illustrating the overlapping downregulated genes in the hippocampus of individuals with PDD, AD and DSD. In the diagram, colors represent the followings; PDD *versus* Control (blue), AD *versus* Control (yellow) and DSD *versus* Control (red).

Analysis of differential gene expression in the hippocampus was performed using five Control, four PDD, six AD, and five DSD samples. In bulk brain tissue, the gene expression profiles can be dramatically influenced by differences in cellular composition. This potential confounder might be due to the variation in grey/white matter ratios introduced during tissue extraction, inter-subject variability or represent disease related alterations^38–40^. To examine the contribution of different sources of biological and technical variations in our dataset, the proportions of major cell-type classes (i.e., astrocytes, endothelia, microglia, neurons, and oligodendrocytes) were first estimated in the samples. This result showed that the cell-type proportions did not differ between the groups (Supplementary Fig. S2). Subsequently, the Kendall’s Tau correlation was calculated between potential sources of variation in our data, such as chronological age (age at death), transcriptional age (RNAAge), PMI, RIN, and sex. The first principal component (PC1) captures the most variance and showed to be negatively correlated with RNAAge, APOE status, and PMI, while it was positively correlated with the RIN value (Supplementary Fig. S3). In addition, chronological age, RNAAge, RIN, PMI, and APOE ε4 status were separately analyzed and plotted against the PC1 and second principal component (PC2). As indicated by a color gradient, mostly RIN values and transcriptional ages showed an opposite but gradual increase in these plots, which suggest that these variables causing the confounding that correlated with the first PC (Supplementary Fig. S4). Further exploration showed that the Variance Inflation Factor for PMI and APOE ε4 status were higher than 5, indicating multicollinearity. To identify genes whose expression level changes in PDD, AD, and DSD, differential gene expression analysis was performed for a total of 24,186 transcripts with sex, RNAAge, and RIN as experimental covariates in Wald tests (see Methods). The exposed differences between the studied groups (i.e., PDD *versus* Control, AD *versus* Control, and DSD *versus* Control) are presented in volcano plots (Fig. 2a). By setting a cutoff value with a false discovery rate (FDR) adjusted *p*-value ≤ 0.05 and |log_2_FC| of 0.3, a total of 2897, 657 and 196 DEGs were identified in the hippocampus of PDD, AD, and DSD individuals, respectively. Each set of DEGs correctly clustered the samples by their group, which is presented in heatmaps (Supplementary Fig. S5). Moreover, common DEGs were identified by a qualitative comparison of the genes that significantly differentiated between each type of dementia with the control group. A total of 29 genes were commonly upregulated, and 16 genes were commonly downregulated among PDD, AD, and DSD samples (Fig. 2b, c). The commonly up- and downregulated genes are listed in Table 2.

**Table 2:** Common up- and downregulated genes in the hippocampus of Parkinson’s disease dementia (PDD), Down syndrome dementia (DSD), and Alzheimer’s disease (AD) individuals.

Gene	Description	Expressed	Chromosome	Transcript length (bp)	ENSEMBL ID	ENTREZ ID
***ARHGEF10L***	Rho guanine nucleotide exchange factor 10 like	Up	Chr1	4625	ENSG00000074964	55160
***HS6ST1P1***	heparan sulfate 6-O-sulfotransferase 1 pseudogene 1	Up	Chr1	1234	ENSG00000187952	N/A
***ADCY5***	adenylate cyclase 5	Up	Chr3	6643	ENSG00000173175	111
***ZNF141***	zinc finger protein 141	Down	Chr4	536	ENSG00000131127	7700
***DCAF16***	DDB1 and CUL4 associated factor 16	Down	Chr4	2633	ENSG00000163257	54876
***CEP44***	centrosomal protein 44	Down	Chr4	600	ENSG00000164118	80817
***TNPO1***	transportin 1	Down	Chr5	11040	ENSG00000083312	3842
***TMEM161B***	transmembrane protein 161B	Down	Chr5	5735	ENSG00000164180	153396
***N4BP3***	NEDD4 binding protein 3	Up	Chr5	5985	ENSG00000145911	23138
***EHMT2***	euchromatic histone lysine methyltransferase 2	Up	Chr6	1517	ENSG00000204371	10919
***FZD9***	frizzled class receptor 9	Up	Chr7	2343	ENSG00000188763	8326
***SH2B2***	SH2B adaptor protein 2	Up	Chr7	490	ENSG00000160999	10603
***SSPOP***	SCO-spondin, pseudogene	Up	Chr7	15799	ENSG00000197558	N/A
***SMARCD3***	SWI/SNF related, matrix associated, actin dependent regulator of chromatin, subfamily d, member 3	Up	Chr7	468	ENSG00000082014	6604
***LOC105376292***	novel transcript	Up	Chr9	1860	ENSG00000227619	105376292
***RALGDS***	ral guanine nucleotide dissociation stimulator	Up	Chr9	5596	ENSG00000160271	5900
***NELFB***	negative elongation factor complex member B	Up	Chr9	2540	ENSG00000188986	25920
***SYT15***	synaptotagmin 15	Up	Chr10	6428	ENSG00000204176	83849
***ARHGAP19***	Rho GTPase activating protein 19	Down	Chr10	5525	ENSG00000213390	84986
***PHRF1***	PHD and ring finger domains 1	Up	Chr11	5278	ENSG00000070047	57661
***ATF7IP***	activating transcription factor 7 interacting protein	Down	Chr12	2298	ENSG00000171681	55729
***ARID2***	AT-rich interaction domain 2	Down	Chr12	735	ENSG00000189079	196528
***MIS18BP1***	MIS18 binding protein 1	Down	Chr14	4577	ENSG00000129534	55320
***SPIRE2***	spire type actin nucleation factor 2	Up	Chr16	663	ENSG00000204991	84501
***DEF8***	differentially expressed in FDCP 8 homolog	Up	Chr16	3620	ENSG00000140995	54849
***GPS2***	G protein pathway suppressor 2	Up	Chr17	1238	ENSG00000132522	2874
***CACNA1G***	calcium voltage-gated channel subunit alpha1 G	Up	Chr17	7497	ENSG00000006283	8913
***BAHCC1***	BAH domain and coiled-coil containing 1	Up	Chr17	10726	ENSG00000266074	57597
***TPGS1***	tubulin polyglutamylase complex subunit 1	Up	Chr19	1114	ENSG00000141933	91978
***LMNB2***	lamin B2	Up	Chr19	769	ENSG00000176619	84823
***CACTIN***	cactin, spliceosome C complex subunit	Up	Chr19	699	ENSG00000105298	58509
***ZNF121***	zinc finger protein 121	Down	Chr19	7177	ENSG00000197961	7675
***ZNF653***	zinc finger protein 653	Up	Chr19	674	ENSG00000161914	115950
***CYP4F3***	cytochrome P450 family 4 subfamily F member 3	Down	Chr19	2078	ENSG00000186529	4051
***ZNF43***	zinc finger protein 43	Down	Chr19	5584	ENSG00000198521	7594
***TTC9B***	tetratricopeptide repeat domain 9B	Up	Chr19	828	ENSG00000174521	148014
***SPTBN4***	spectrin beta, non-erythrocytic 4	Up	Chr19	6105	ENSG00000160460	57731
***GRIN2D***	glutamate ionotropic receptor NMDA type subunit 2D	Up	Chr19	5511	ENSG00000105464	2906
***ZNF615***	zinc finger protein 615	Down	Chr19	4094	ENSG00000197619	284370
***TRPM2***	transient receptor potential cation channel subfamily M member 2	Up	Chr21	5989	ENSG00000142185	7226
***HIRA***	histone cell cycle regulator	Up	Chr22	3395	ENSG00000100084	7290
***PRR5***	proline rich 5	Up	Chr22	1726	ENSG00000186654	55615
***RAP2C***	RAP2C, member of RAS oncogene family	Down	ChrX	3341	ENSG00000123728	57826
***MT-TC***	mitochondrially encoded tRNA-Cys (UGU/C)	Down	MT	66	ENSG00000210140	N/A
***MT-TY***	mitochondrially encoded tRNA-Tyr (UAU/C)	Down	MT	66	ENSG00000210144	N/A

The genes were ranked based on their chromosomal location.

**Fig. 3:**
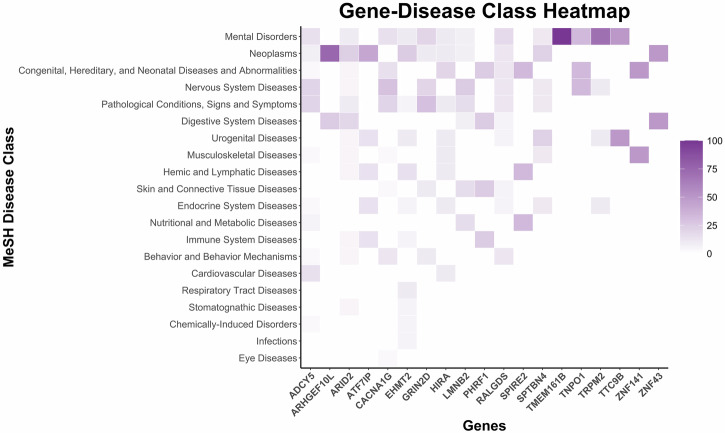
Gene-Disease associations of differentially expressed genes (DEGs) in dementia. Common DEGs identified in individuals with Parkinson’s disease dementia (PDD), Alzheimer’s disease (AD), and Down syndrome dementia (DSD) were queried in the DisGeNET curated database. Heatmap of Gene-Disease class associations for shared DEGs across PDD, AD, and DSD, organized according to the Medical Subject Headings (MeSH) disease classification. Color intensity reflects the level of gene expression or activity.

**Fig. 4:**
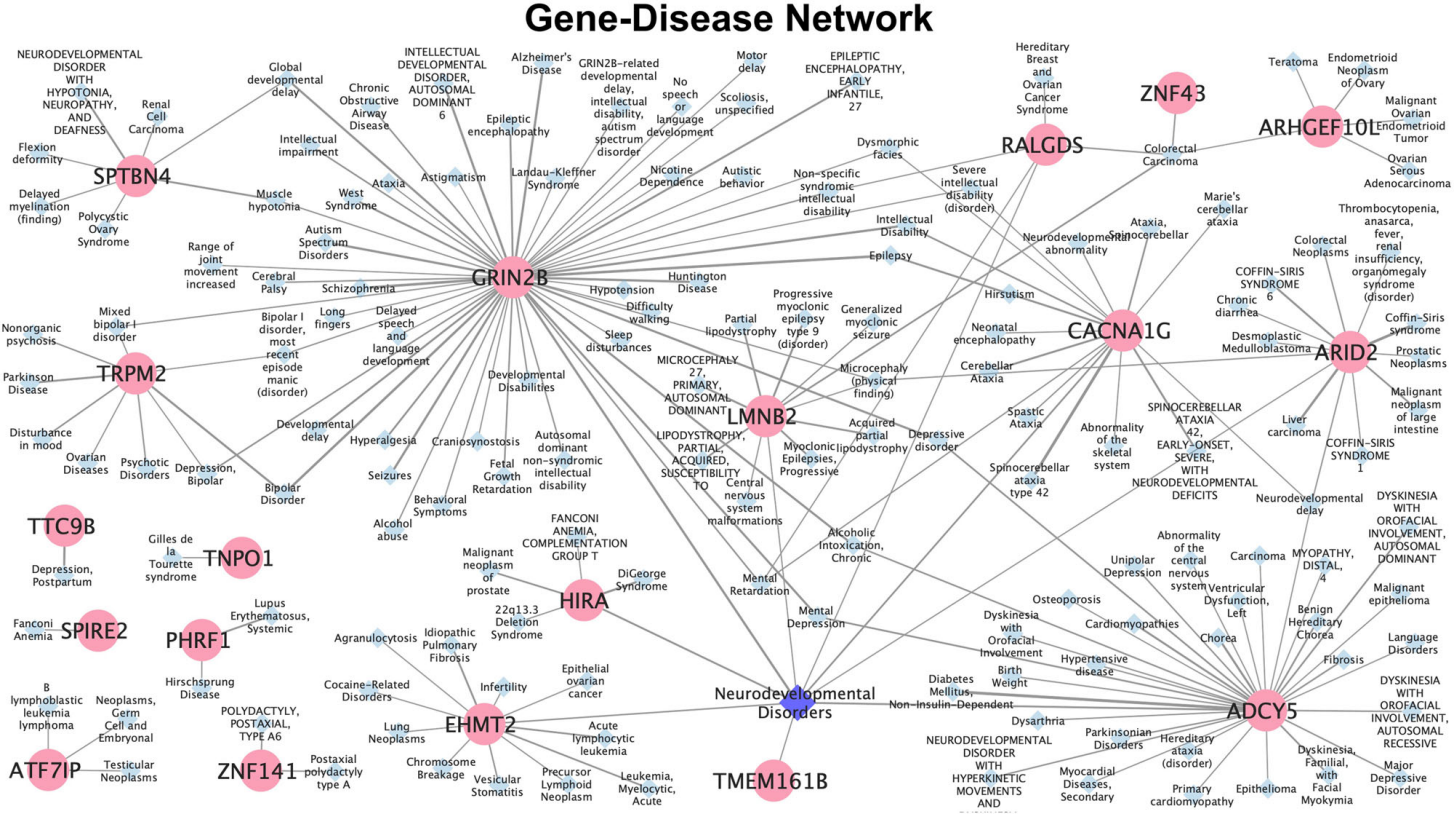
Gene-Disease network of differentially expressed genes (DEGs) in dementia. Gene-Disease network representing associations between the common DEGs in individuals with Parkinson’s disease dementia (PDD), Alzheimer’s disease (AD), and Down syndrome dementia (DSD) and their corresponding diseases. Blue nodes represent diseases and pink nodes represent genes, while the thickness of the edges denoting the associations between the gene and the disease.

Next, a search was performed with the common DEGs using an expert-curated database (DisGeNET), which covers information on Mendelian and complex diseases (Fig. 3). This database prioritized and only supported gene-disease associations for *ADCY5* (adenylate cyclase 5), *ARHGEF10L* (Rho guanine nucleotide exchange factor 10 like), *ARID2* (AT-rich interaction domain 2), *ATF7IP* (activating transcription factor 7 interacting protein), *CACNA1G* (calcium voltage-gated channel subunit alpha1 G), *EHMT2* (euchromatic histone lysine methyltransferase 2), *PHRF1* (PHD and ring finger domains 1), *RALGDS* (ral guanine nucleotide dissociation stimulator), *GRIN2D* (glutamate ionotropic receptor NMDA type subunit 2D), *HIRA* (histone cell cycle regulator), *LMNB2* (lamin B2), *SPIRE2* (spire type actin nucleation factor 2), *SPTBN4* (spectrin beta, non-erythrocytic 4), *TMEM161B* (transmembrane protein 161B), *TNPO1* (transportin 1), *TRPM2* (transient receptor potential cation channel subfamily M member 2), *TTC9B* (tetratricopeptide repeat domain 9B), *ZNF141* (zinc finger protein 141), and *ZNF43* (zinc finger protein 43). The top 5 disease associations were mental disorders, neoplasms, congenital, hereditary, and neonatal disease and abnormalities, nervous system disorders, and pathological conditions, signs and symptoms (Fig. 3), as described with the comprehensive controlled vocabulary of Medical Subject Headings (MeSH). Furthermore, the gene associated with the most diseases was *GRIN2B* (52) followed by fewer disease associations for *ADCY5* (32), *CACNA1G* (17), *ARID2* (13), *EHMT2* (12), and *LMNB2* (11) (Fig. 4). Although some other diseases were related with the common DEGs, the majority of associated diseases were related to neurological disorders. Interestingly, most genes (i.e., *GRIN2B*, *CACNA1G*, *ADCY5*, *LMNB2*, *EHMT2*, *ARID2*, *HIRA*, *RALGDS*, and *TMEM161B*) were associated with each other through neurodevelopmental disorders, which is labeled as a class for Mental Disorders in the DisGeNET database.

### Gene ontology reveals common molecular functions and cellular components among the dementias

**Fig. 5:**
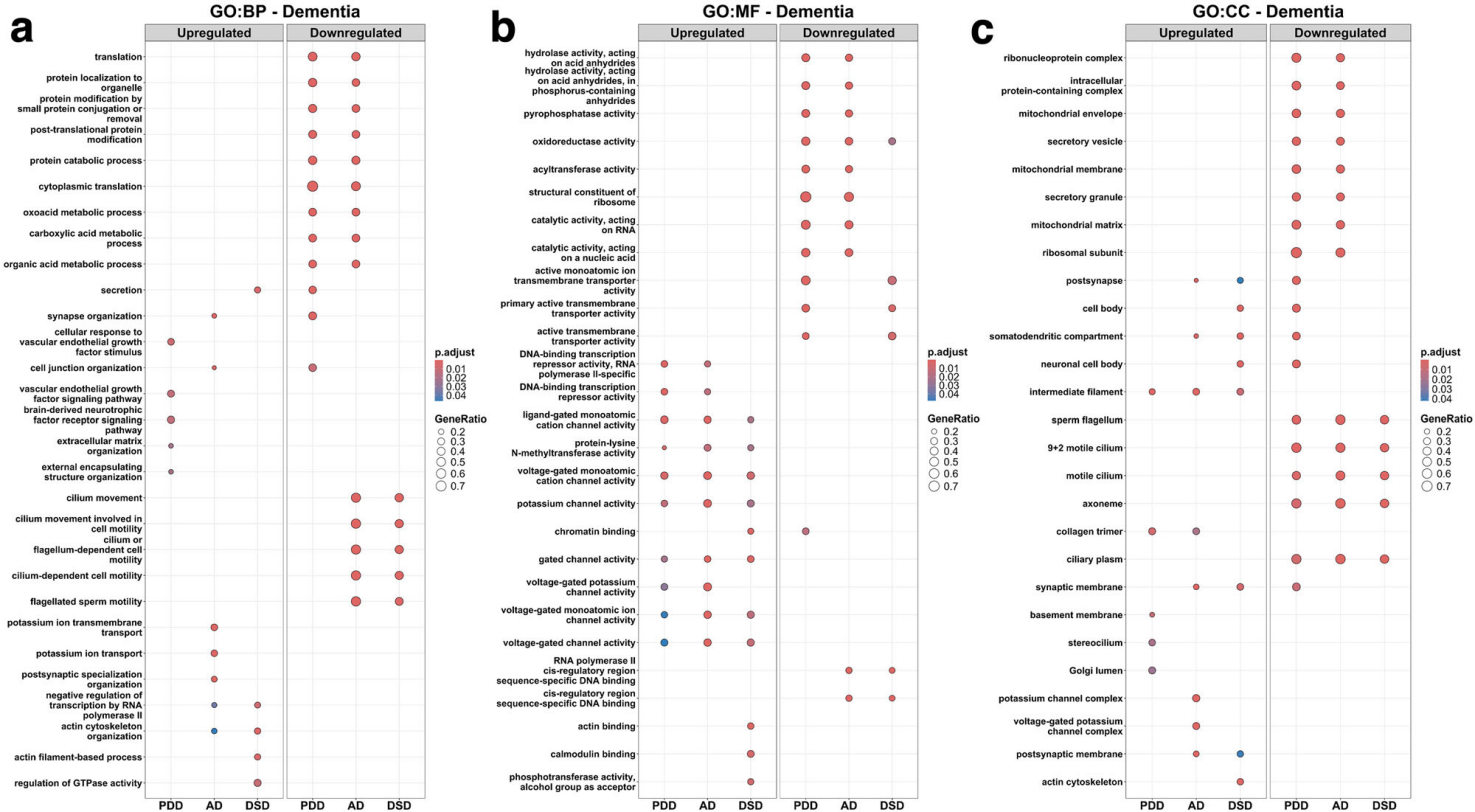
Gene Ontology (GO) enrichment analysis for DEG in dementia. GO was performed through a gene set enrichment analysis for individuals with Parkinson’s disease dementia (PDD), Alzheimer’s disease (AD), and Down syndrome dementia (DSD). **a** Biological processes (BP) that were significantly enriched or impaired. **b** Molecular functions (MF) that were significantly enriched or impaired. **c** Cellular components (CC) that were significantly enriched or impaired. In each plot, dot size indicates the number of genes in that gene set by ratio (GeneRatio) and dot color reflects statistical significance based on FDR-adjusted p-values (p.adjust).

Gene Ontology (GO) analysis did not detect a commonly enriched or impaired biological process (BP) term among the different types of dementia (Fig. 5a). The most overlapping enriched terms for molecular function (MF) were related to voltage-gated channel, ligand-gated channel, and protein-lysine *N*-methyltransferase activities. In contrast, the oxidoreductase activity was impaired in PDD, AD, and DSD (Fig. 5b). The enriched terms for cellular components (CC) were mainly associated with intermediate filaments, whereas commonly impaired CC terms corresponded with cilia and flagella structures (Fig. 5c). Disease Ontology (DO) analysis associated DSD with characteristic terms (i.e., genetic disease and chromosomal disease), indicating an appropriate and accurate transcriptomic analysis. However, no single term was shared among the dementias in DO, while oxidative phosphorylation was the only term commonly impaired in the Kyoto Encyclopedia of Genes and Genomes (KEGG) analysis among the different types of dementia (Supplementary Fig. S6).

### Chromatin organization module is altered in dementia

**Fig. 6:**
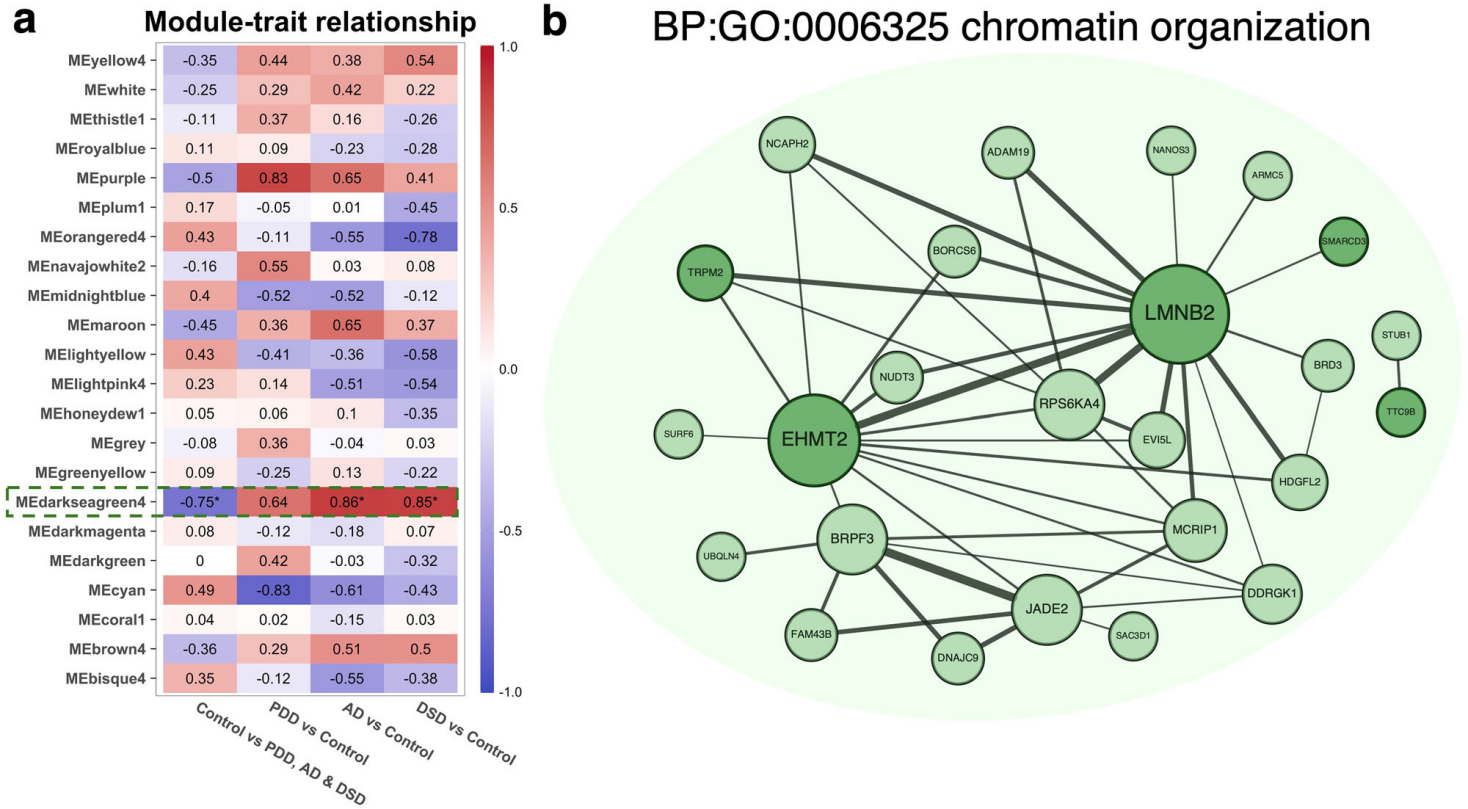
Identification and network visualization of the darkseagreen4 module. **a** Heatmap of module-trait correlation across non-demented controls (Control), Parkinson’s disease dementia (PDD), Alzheimer’s disease (AD), and Down syndrome dementia (DSD). The darkseagreen4 showed a strong negative correlation with the control (non demented) cases (*p*-value = 0.003), while this association was reversed in PDD (*p*-value = 0.067), AD (*p*-value = 0.017) and DSD (*p*-value = 0.041). Statistical significance was based on the FDR-adjusted p-values derived from the Pearson correlation test, which was presented with *p-value ≤ 0.05. **b** The co-expression network of the darkseagreen4 module included 70 genes, where only the most strongly intra-connected genes are shown. Functional enrichment analysis showed that this module was significantly associated with chromatin organization (GO:0006325). Edges represent gene-gene correlation, while node size indicates number of connections (degree) for each gene. Darker green nodes represent the genes that were commonly upregulated across all dementia groups compared to the control group.

For the weighted gene co-expression network analysis (WGCNA), the whole dataset was used to construct an adjacency matrix. Then, a network was constructed that was in line with the characteristics of a scale-free network. Therefore, the soft thresholding power (β) was set to seven to ensure a correlation coefficient above 0.85 (Supplementary Fig. S7a). The WGCNA R package was used to construct the co-expression network module and visually display the modules’ gene correlation. After merging different modules based on their similarities, a total of 22 co-expression modules were obtained with at least 50 genes in each module (Supplementary Fig. S7b). A heatmap was created based on module-trait relationship (Fig. 6a), according to the Spearman correlation coefficient to evaluate the association between each module and the sample group (i.e., Control, PDD, AD, and DSD). Among the modules, darkseagreen4 (*p*-value ≤ 0.05) showed a high negative correlation with the non-demented samples (i.e., Control), while this association was reverted for the groups with demented individuals (i.e., PDD, AD, and DSD) compared to the Control. The genes of the darkseagreen4 module were selected for GO analysis, resulting that these genes were mainly associated with the biological process of chromatin organization (BP:GO:0006325; *p*-value = 0.0002). Other significant GOs associated biological processes with this module were protein-DNA complex organization (BP:GO:0071824; *p*-value = 0.0004), chromatin remodeling (BP:GO:0006338; *p*-value 0.0005), and regulation of DNA metabolic process (BP:GO:0051052; *p*-value = 0.0247). In addition, GO analysis showed that the darkseagreen4 module was significantly associated with various molecular functions, including histone H3K14 acetyltransferase activity (MF:GO:0036408; *p*-value = 0.0150), histone H4K8 acetyltransferase activity (MF:GO:0043996; *p*-value = 0.0229), histone H4K5 acetyltransferase activity (MF:GO:0043995; *p*-value = 0.00229), histone H4K12 acetyltransferase activity (MF:GO:0043997; *p*-value = 0.0274), histone modifying activity (MF:GO:0140993; *p*-value = 0.0301), and histone H3 acetyltransferase activity (MF:GO:0010484; *p*-value = 0.0378).

To explore gene-gene interactions, the edges and nodes (threshold 0.1) of this module were exported and visualized in Cytoscape (Fig. 6b). Interestingly, a relatively high number of commonly upregulated genes among the dementias (5/29 = 17%) were present in the darkseagreen4 module (70 genes). Two of these genes (*EHMT2* and *LMNB2*) shown to be hub genes and were upregulated across the three types of dementia (Supplementary Fig. S8). More specific, the effect size of *EHMT2* in log_2_FC was 0.4134, 0.4236, and 0.6005 for PDD, AD, and DSD, respectively. The other hub gene, *LMNB2*, presented an effect size of 0.5303, 0.4857, and 0.5373 log_2_FC in PDD, AD, and DSD, respectively. Overall, the aberrant overexpression of these two hub genes represents that they play a pivotal role in the disruption of chromatin structure within individuals with PDD, AD, and DSD.

## Discussion

In line with previous studies, we found that the biological age was significantly accelerated in DS individuals compared to non-demented controls^35,41^. Overlapping transcriptomic analysis identified 45 commonly dysregulated genes (i.e., 29 up- and 16 downregulated) across PDD, AD, and DSD. For these neurodegenerative dementias, the top overlapping functional enriched terms were protein-lysine *N*-methyltransferase activity, intermediate filament, and voltage-gated channel activity. WGCNA identified a module associated with chromatin organization that showed a strong negative correlation with control (i.e., non-demented) samples, whereas this association was even reversed in the dementia groups. This resulted in the identification of two hub genes: euchromatic histone lysine methyltransferase 2 (*EHMT2*) and lamin B2 (*LMNB2*), which were also common DEGs across the dementias. Previous studies have associated dysregulation of these genes with specific neurodegenerative and developmental disorders, while our findings extend their potential relevance across multiple dementias^42–44^.

Neurodegenerative dementias are expected to be a major contributor to the global burden of disease. Hence, gaining more knowledge through transcriptomic studies to infer the pathogenic mechanisms involved will be key in addressing the expected increase in the number of individuals affected by dementia^2^. The strongest risk factor to develop dementia is aging, which might be better represented through people’s biological age (i.e., accumulation of cellular damage over time) than by one’s chronological age^45^. Many studies have already demonstrated accelerated aging in DS using algorithms based on various biomarkers, such as Horvath’s epigenetic clock, GlycoAgeTest, brain predicted age, and IgG-glycans^35,41,46,47^. The application of each algorithm depends on the availability of biological material or aim of the study. For instance, the predicted biological age based on DNA methylation cannot be directly correlated with the transcriptional age, as the exact regulatory role of methylation on the transcriptome is not completely uncovered. Therefore, we inferred the accelerated aging for hippocampal tissues from three neurodegenerative dementias based on transcript levels using an RNA-based algorithm, which was specifically trained for human brain tissue with a high sample size and over 1,500 age-related genes^32^. Our research estimated a significant increase of biological (or transcriptional) age compared to their chronological age in all dementia types, but not in non-demented control cases. The accelerated age in the hippocampus of DSD individuals was significantly expedited between 20.3–45.2 years. Accelerated transcriptional aging has not only been shown to be disease-specific but to differ between brain regions and genetic backgrounds as well^32,35,48^. Consistent with our DSD results, Murray and colleagues observed an accelerated age of 20.4–31.1 years in DS individuals from the United Kingdom, whereas this age shift remained constant throughout their lifespan^35^. Together with our findings, this suggests that mainly DS is responsible for the accelerated aging and not dementia or only to a certain degree. Of note, accelerated transcriptional aging in DSD was independently confirmed with the BiT age clock algorithm, which is a transcriptomic-based aging clock that uses binarization of *C. elegans* transcriptomes to define a gene set that predicts the biological age^37^. Importantly, the transcriptional age negatively correlated with the PC1, indicating it potentially affects the downstream transcriptomic analysis. Altogether, this led to incorporation of the transcriptomic ages as one of the covariates in our negative binomial generalized linear model to identify differential expressed transcripts between the dementias and non-demented cases, as the chronological age possibly did not reflect disease-specific aging processes.

Cell composition and RNA quality have also been shown to be major confounders in transcriptomic studies^8,49^. Although we obtained high RIN values (5.8–9.2) from human post-mortem brains, these numbers significantly correlated with the PC1 values and were negatively correlated with the RNAAge, presenting an inverse relationship captured by PC1. This indicated that the transcriptional age and RIN have a strong but opposite contribution by capturing the variance and, thus, were incorporated in our gene expression model. The cell-type proportions were estimated with the MuSiC2 deconvolution algorithm^50^. In this study, there was no differences detected in cell-proportions between the dementias and control cases. Cell-type deconvolution depends on gene expression profiles, whereas cell composition differences in our neurodegenerative conditions may have been minimized through the implementation of aged controls in our study design^51^. Previous studies have shown changes in cell-type composition between DS and normal control brain tissues^52,53^. However, the direction of deregulation for certain cell-types was conflicting between these studies, indicating the limitations of these algorithms. Single-cell RNA-sequencing (scRNA-seq) and single-nucleus RNA-sequencing (snRNA-seq) methods might overcome the limitations of using cell-type estimation algorithms in bulk RNA-seq. Nevertheless, it is challenging to perform scRNA-seq in brain tissues due to the complex network of axons, dendrites, and glia that are lost and/or damaged after tissue dissection and cell dissociation^54^. In post-mortem brain tissues, snRNA-seq can be currently performed, however, at a cost of 50–80% transcriptomic reduction, which involves the complete loss of relatively low expressed transcripts^55^. Therefore, we used the scRNA-seq dataset from Darmanis et al. to estimate cell-type proportions in our bulk brain tissues, allowing us to detect also the “dark transcriptome” (i.e., transcripts localized away from cell bodies) that scRNA-seq does not take into account^54^.

Bulk RNA-seq was performed together with the ribosomal RNA depletion method, which allows sequencing of both coding and all non-coding transcripts. This approach has shown to result in a higher transcript coverage with unique transcriptome features compared to polyA+ selection methods in human post-mortem tissue^49,56^. For instance, we identified the upregulation of *LOC105376292* (i.e., a novel non-coding RNA transcript) and linked its potential involvement in neurodegenerative dementias, while a genome-wide significant locus for this transcript was recently associated with the brain arterial diameter (i.e., a biomarker for cerebrovascular disease, cognitive decline, and dementia) within a European population^57^. We acknowledge that the sample size for RNA-seq was small given the challenges associated with hippocampal tissue acquisition. There were also fewer males in the non-demented control group compared to the AD and DSD groups. Although these differences between the groups were not statistically significant and sex was included as a covariate in the analysis, the identification of DEGs in this study might still be biased towards one sex. Nonetheless, the unique combination of samples from different brain banks in this study allowed us to identify common transcriptional changes between potentially overlapping neurodegenerative dementias. In addition, the standardized neuropathological examination by experts ensured us with excellent and high-quality samples.

Our transcriptomic analytic pipeline highlighted *LMNB2* (lamin B2) and *EHMT2* (euchromatic histone lysine methyltransferase 2) as hub genes in a chromatin organization module and showed them to be commonly upregulated transcripts in hippocampal tissue among the neurodegenerative dementia types. The intermediate filament protein LMNB2 plays a part in the formation of the nuclear lamina and the regulation of cellular processes, such as tissue development, cell cycle, cell proliferation, apoptosis, chromatin localization and stability, and DNA methylation. The influence of abnormal expression and mutations of LMNB2 has been gradually discovered in laminopathies and cancers^58^. Similarly, EHMT2 (also known as G9a) has been indicated to be involved in cell proliferation, apoptosis, cell invasion, and DNA methylation in neuroblastoma, a childhood neoplasm arising from neural crest cells^59^. Rapidly progressive dementia can be caused by certain types of cancers, which was also suggested in our gene-disease association study with the annotation of neoplasms as one of the highest MeSH class^60^.

The main purpose of lamin B2 is to preserve nucleolus organization and stabilize nucleolin within the nucleolus, which is a nuclear compartment and is the site of ribosomal DNA transcription, processing, and ribosome biogenesis^61^. Over 40% of heterochromatin has shown to be associated with the nucleolar periphery, leading to the formation of nucleolus-associated chromatin domains^62^. These domains are enriched with heterochromatin from (peri)centromeric chromosomal regions and contain mostly repressive chromatin marks, such as dimethylation at lysine 9 of histone H3 (H3K9me2). Those regions can rearrange their configuration due to lamin levels, leading to a dynamic three-dimensional genomic architecture^63^. Our co-expression network analysis suggests *LMNB2* to be a key player in chromatin organization. Defects in lamins A and C have been involved and classified as laminopathies, including muscular dystrophy, and progeria. Moreover, lamin B1 or B2 alteration has previously been linked to various neuropathies^64^. Consistent with our results, Gil and colleagues observed an increase of LMNB2 levels in pyramidal hippocampal neurons of AD patients at Braak stages V-VI, which was related with nucleoli displacement to the periphery and signs of neuronal attrition^44^. An AD model presented deregulation of lamin B that led to aberrant nucleo-cytoskeletal coupling and promoted heterochromatin relaxation and neuronal death, suggesting AD can be considered as an acquired neurodegenerative laminopathy linked to aging^65^. Furthermore, aneuploid chromosomes have been shown to be mis-localized in cell populations with depleted lamin B2, but not for other lamin subtypes, indicating together with our data a role for LMNB2 in trisomy 21 as well^66^. Nevertheless, the role of *LMNB2* in dementia should be further explored with functional studies in models for PD, AD, and DS.

The other hub gene, EHMT2, has recently been associated with PD in the European population through a genome-wide association study^67^. This methyltransferase specifically targets H3K9me2, which is associated with transcriptional gene repression^68^. Histone methylations have shown to be involved in the dysregulation of synaptic functions and associated with mental disorders, which was also the highest annotated MeSH class in this study^69,70^. However, future experimental studies (e.g., western blotting or immunohistochemistry) should validate the increase of H3K9me2 levels in the types of neurodegenerative dementias. Inhibition of upregulated *EHMT2* levels has previously shown to decrease H3K9me2 levels, restore synaptic functions, prevent neuronal death, and rescue motor impairment without affecting the formation of α-synuclein in a mouse model for PD^42^. In a late-stage AD mouse model, the increase of *EHMT2* expression led to augmented H3K9me2 levels, but not for this model at an early-stage, suggesting an age dependence of this epigenetic change happening later in life. Correspondingly, the inhibition of this methyltransferase rescued synaptic and cognitive functions, but failed to reduce the amyloid load in those AD mice^43^. In line with previous reports, our findings suggest EHMT2 dysregulation may play a central role in neurodegenerative dementias and highlight this methyltransferase as a potential therapeutic target warranting further experimental validation. Interestingly, different pharmaceutical interventions have been explored to alleviate cognitive impairment in DS, although with a limited success in clinical trials^71,72^. Until now, we are the first study that links *EHTMT2* dysregulation with DSD, which suggests exploring the beneficial effects of EHMT2 inhibitors in preclinical DS models and to potentially ameliorate their cognitive impairment and synaptic dysfunction during adult life stages^73^.

Although promising, our findings should still be interpreted with caution, as the study is limited by the modest sample size and the constraints of bulk RNA-seq. Nevertheless, the consistent signals observed among the different dementia groups suggest common molecular processes that may contribute to hippocampal vulnerability. Future studies using single-cell and spatial transcriptomic approaches will be essential to validate the role of these candidate genes and to determine whether they represent viable biomarkers or therapeutic targets. Overall, the transcriptomic analysis pipeline and unique approach presented in this work provides an original strategy to discover novel biomarkers or disease-altering targets in potentially overlapping neurological diseases and a promising research avenue for other diseases.

## Methods

### Ethical approval

This study was approved by the Ethics and Deontology Committee of the ICM Paris Brain Institute (COMETH-ICM). Brain tissue specimens are rare and precious and originate from donations for research purpose. The donors have signed an informed consent for research into diseases of the nervous system. The Biomedical Research Law (Law 14/2007, of July the 3rd, Biomedical Research) and (Royal Decree that developed after the Biomedical Research Law) were passed to regulate the proper collection, storage and use of biological samples of human origin, and to promote their use for biomedical research by following good ethical and scientific practices. All experiments were performed in accordance with the Declaration of Helsinki.

### Sample selection

Post-mortem brain tissue of PDD, AD, and DSD individuals were pathologically confirmed and obtained from four European brain banks: National Brain Bank Neuro-CEB, Pitié-Salpêtrière Hospital (Paris, France); Institute of Psychiatry, King’s College London Brain Bank (London, United Kingdom); Cambridge Brain Bank, Addenbrooke’s Hospital, Cambridge University Hospital (Cambridge, United Kingdom), and the Neurobiobank of the Institute Born-Bunge (Antwerp, Belgium). The cohort included five control cases (Control) from non-demented individuals who died without known neurological disorders, four samples from PDD patients, six samples from patients with sporadic AD, and five samples from DSD individuals with neuropathological signs of AD, including NFT and Aβ-containing senile plaques at histological examination (Supplementary Table S1)^74^. The samples were collected at autopsy and stored at -80°C until further processing.

### Sample preparation

Dissected hippocampi were weighted to calculate the homogenization volume of the buffer for obtaining a 20% concentrated suspension (weight/volume). The tissue was homogenized in ice-cold 50 mM Tris–HCl (pH 7.4) supplemented with Halt^™^ Protease and Phosphatase Inhibitor Cocktail (#78438, Thermo Fisher Scientific, Pittsburgh, PA, United States) by using the Bio-Gen PRO200 Homogenizer (#01-01200, PRO Scientific Inc., Oxford, CT, United States) at setting three for 30 s and then at full speed for one minute. Subsequently, the suspension was centrifuged at 3000 x g for 10 min (4 °C). Then, the supernatant was used for RNA extraction. Total RNA was isolated with RNeasy Mini Kit (#74104, Qiagen, Hilden, Germany), according to manufacturer’s instructions to achieve maximum yields of RNA. Next, the samples were treated with the Heat&Run® gDNA Removal Kit (#80200, ArticZymes, Tromsø, Norway) to avoid amplification of genomic DNA during further processing steps. The RNA concentration and purity were analyzed using the NanoDrop-1000 Spectrophotometer (Thermo Fisher Scientific, Pittsburgh, PA, United States). For each sample a total of 42–336 ng was obtained. The RIN was assessed using the RNA 6000 Pico Kit of Bioanalyzer 2100 system (#5067-1513, Agilent Technologies, Santa Clara, CA, United States). All RNA samples were stored at −80 °C until further processing.

### RNA-sequencing and data quality control

A total of 10 ng RNA was used for downstream RNA-seq application. First, the SMARTer® Stranded Total RNA-Seq Kit v3 - Pico Input Mammalian (#634485, Takara Bio, Kusatsu, Japan) was used for library preparation, which was followed by a purification with AMPure XP beads (#A63880, Beckman Coulter Inc., Brea, CA, United States). Then, library fragments originating from rRNA (18S and 28S) and mitochondrial rRNA (m12S and m16S) were cleaved by ZapR v3 in the presence of mammalian specific R-Probes v3 (Takara Bio, Kusatsu, Japan). Paired-end sequencing for 50 bp each was performed on the NovaSeq 6000 platform (Illumina Inc., San Diego, CA, United States) to a depth of around 50 million reads (i.e., 25 million reads paired-end fragments). FASTQ output was assessed using MultiQC (version 1.10.1) with default settings prior to alignment and quantification. Quality was visually inspected to observe the “sequence quality”, “per tile sequencing quality”, “overrepresented sequences”, “adapter content” and other quality parameters^75^.

### RNA expression quantification and filtering

Raw sequencing reads in the FASTQ files were mapped with STAR (version 2.7.8a) against the Gencode v41 transcriptome, which was based on the GRCh38.p13 reference genome^76^. BAM files were deduplicated with UMI-Tools dedup (version 1.1.2) using the --method=unique to retain one representative read per unique UMI. The generation of a table of counts with the subread R package (version 2.0.3)^77^. The options applied to quantify the abundance at gene level were -p to count read pairs, -t “exon” as features to be quantified, --largestOverlap to assign reads to the feature with the largest overlap and -g “gene_name” to collapse transcript-level quantification into gene-level counts.

### Deconvolution

The raw count matrix obtained from STAR was imported in RStudio (version 2025.05.1 + 513) for performing bulk RNA-seq deconvolution using the MuSiC2 algorithm^50^. First, this matrix was converted to counts per million (cpm) by dividing with the total number of reads and multiplying by 10^^6^. An ExpressionSet was created with the converted matrix and the single cell RNA sequencing (scRNA-seq) reference dataset from Darmanis et al. was imported through the scRNAseq R package (version 2.22.0)^78^. Subsequently, the scRNA-seq reference dataset was filtered for astrocytes, endothelia, microglia, neurons, and oligodendrocytes. The proportions of these cell-types for each sample were estimated with the function music2_prop_t_statistics from MuSiC2 (version 0.1.0)^50^. Then, a one-way ANOVA followed by the Tukey’s HSD multiple-comparisons *post hoc* test was performed to assess significance for each cell-type between the Control, PDD, AD and DSD individual groups (Supplementary Fig. S2).

### Transcriptional age calculation

The “predict_age” function of the RNAAgeCalc R package (version 1.20.0) was used to compute the transcriptional age for each RNA sample^32^. First, the transcript length for all transcripts were downloaded using the biomaRt R package (version 2.64.0) with Ensembl 114. Then, the Fragments Per Kilobase of exon per Million mapped reads (FPKM) was calculated by first dividing the raw counts matrix by the library size and then by gene length (i.e., transcript length). Finally, this FPKM counts matrix was provided to the “predict_age” function by setting the parameters as follows: exprtype “FPKM”, tissue “brain”, idtype “SYMBOL”, stype “caucasian”, signature “DESeq2” and maxp “30000”.

Independently, the transcriptional age was computed with BiT age software, using 141 human predictor genes^37^. Therefore, the raw count matrix was converted to cpm by dividing with the total number of reads and multiplying by 10^^6^. Then, the options applied to compute the transcriptional age with the BiT age software were –intercept “79.2”, --with “correction”, --median-age “50”, --sd-age “10”.

### Covariate selection

Sources of variation in the RNA-sequencing data were identified using principal component analysis (PCA) performed on gene-level expression filtered based on cpm values > 0.33 in at least 6 samples (24,186 genes). The filtered genes were normalized using Trimmed means of M values (TMM) method of the edgeR R package (version 4.6.3), which is a statistical package based on generalized linear models^79,80^. The transcriptional age (RNAAge), RIN, PMI and APOE ε4 status were significantly correlated with the first PC (Supplementary Fig. S3). Samples were plotted by their first two principal components to determine how well the group (i.e., Control, PDD, AD, DSD), chronological age, transcriptional age, RIN, PMI, and APOE ε4 status separated (Supplementary Fig. S4). Of note, none of the estimated cell proportion for each cell-type significantly differed between groups (see section deconvolution), resulting that those proportions were not included into the linear model as a covariate to adjust for cell composition variation. Moreover, PMI and APOE ε4 status suggested multicollinearity with other covariates in the model. Finally, the model for differential expression consisted of the groups (i.e., Control, PDD, AD, and DSD) and the covariates RNAAge, RIN, and sex. Although the sex difference was not significant between groups due to a small sample size, it was included as a covariate because the AD and DSD groups contained three times more males as the Control group. This was to exclude a bias for detecting DEGs due to their location on sex chromosomes.

### Differential gene expression

Differential gene expression of the RNA-sequencing was assessed on gene expression level filtered based on cpm values > 0.33 in at least 6 samples (24,186 genes), using DESeq2 R Package (version 1.48.1)^81^. The Wald test was used to detect DEGs in a pairwise manner (i.e., PDD *versus* Control, AD *versus* Control, and DSD *versus* Control), controlling for covariates identified previously (see section 2.7). Prior to correction, covariates to be used in the model were scaled to ensure that continuous variables that are measured on different scales (i.e., RIN *versus* RNAAge) are comparable. Significant genes were obtained with a correction for FDR adjusted p-value ≤ 0.05 and |log_2_FC| of 0.3, which was identical to the settings from a recent bulk RNA-seq study performed with human DS hippocampal tissues^53^. The volcano plots and heatmaps (Supplementary Fig. S5) depicting the DEGs were created with the ggplot2 (version 3.5.2) and pheatmap (version 1.0.13) R packages, respectively.

### Functional analysis

Gene Set Enrichment Analysis (GSEA) was performed on the GO, DO and KEGG databases of a pre-ranked list according to the log_2_FC x -log_2_(p-value) metric, which penalize large fold changes that have large (non-significant) *p*-values. GSEA was used to test for enrichment of specific gene sets within the ranked list to define whether specific signaling pathways were enriched among upregulated or downregulated genes^82^. GO enrichment was performed to investigate the gene-related BP, MF, and CC. DO enrichment analysis was used to explore genes-related diseases. KEGG enrichment analysis was conducted to explore gene-related signaling pathways. The obtained results were visualized with the clusterProfiler R package (version 4.16.0)^83^. Statistical significance was set at an FDR-adjusted p-value ≤ 0.05. In addition, an enrichment analysis was performed with the common demented DEGs (45 genes) using the DisGeNET R package (version 1.2.5), which has genes’ association curated data to disease and phenotypic traits^84,85^.

### Weighted gene co-expression network analysis

To screen for a potential group of genes that are specifically associated with dementia, the WGCNA R package (version 1.73) was used to perform a WGCNA to find modules in demented individuals and non-demented controls^86^. First, the raw counts matrix was filtered based on counts < 15 in more than 75% of the samples (23,150 genes). Subsequently, the Pearson correlation coefficient was calculated between each pair of genes to evaluate the expression similarity of genes and acquire a correlation matrix. The soft threshold function was applied to convert the correlation matrix into a weighted neighborhood matrix. An optimal soft power threshold of seven was selected through the soft connectivity algorithm, ensuring that the gene correlations were maximally consistent with the scale-free topology and a negative slope. Thereafter, a topological overlap matrix (TOM) was constructed from the adjacency matrix. The TOM was hierarchically clustered using average linkage hierarchical clustering with “1-TOM”, which is a dissimilarity measure (dissTOM). The TOM indicates how similar two genes are in terms of their connectivity in the network (i.e., network interconnectedness), helping identify robust, biologically meaningful modules of co-expressed genes. Modules were defined as branches of a dendrogram and derived through the dynamic tree cutting method, applying a minimum module size of 50 and a maximum deep split. These initially generated modules were merged based on module eigengenes, using correlation-based adjacency as dissimilarity matrix. Furthermore, the modules with a smaller distance of less than 0.25 were merged into a single module. Each module was summarized by module eigengene (ME), representing the characteristic expression profile. Module-trait associations were computed with biological traits (i.e., Control, PDD, AD, DSD) and significant correlation indicated potential key modules, where key modules’ genes were considered key genes. The edges and nodes parameters were derived for significant modules in the module-trait associations and imported in Cytoscape (version 3.10.3)^87^. Key genes presented in these modules were used for generating an interaction network, gene ontology analysis and identification of hub genes. Finally, target genes were inferred through the intersection of DEGs with key modules’ genes.

## Supplementary information

Supplementary Information

## Data Availability

The RNA-seq data that support the findings of this study have been deposited in the Gene Expression Omnibus repository with the series record GSE318560.

## Code availability

The R code and instructions for full reproduction of the results are available at https://github.com/renealbertjohan/NPJ_dementia.
