# Supplementary material for: Common pathogenic mechanisms in the hippocampus across neurodegenerative dementias: Alzheimer’s disease, Down syndrome, and Parkinson’s disease
Source: NPJ Dement. 2026 Apr 29;2(1):32. doi: 10.1038/s44400-026-00075-x (PMC13128439; doi:10.1038/s44400-026-00075-x)
Supplement: Supplementary file 1 — Supplementary Information [file 44400_2026_75_MOESM1_ESM.pdf]

Supplementary material

Case #	Clinical diagnostic	AD Braak	Thal phase	CERAD score	PD Braak	Sex	Age	PMI	ApoE	Brain bank
No. 1	Control	I/II	0	None	*N/A*	F	73	1.50	ε3/ε3	NBB-IBB
No. 2	Control	I/II	0	None	*N/A*	F	81	2.25	ε2/ε3	NBB-IBB
No. 3	Control	*N/A*	*N/A*	*N/A*	*N/A*	F	81	5.53	ε3/ε3	NBB-IBB
No. 4	Control	*N/A*	*N/A*	*N/A*	*N/A*	F	89	2.50	ε3/ε3	NBB-IBB
No. 5	Control	I/II	1-2	None	*N/A*	M	78	2.00	ε3/ε3	NBB-IBB
No. 6	PDD	V	4	*N/A*	V/VI	F	68	24.00	ε4/ε4	NeuroCEB
No. 7	PDD	VI	*N/A*	*N/A*	V/VI	F	85	21.00	ε3/ε4	NeuroCEB
No. 8	PDD	I/II	*N/A*	*N/A*	III	F	86	3.25	ε3/ε3	NBB-IBB
No. 9	PDD	III	*N/A*	*N/A*	I/II	M	80	8.00	ε3/ε3	NBB-IBB
No. 10	AD	IV/V	4-5	Moderate	*N/A*	F	68	16.50	ε3/ε4	NBB-IBB
No. 11	AD	V/VI	4-5	Frequent	*N/A*	F	70	16.50	ε4/ε4	NBB-IBB
No. 12	AD	VI	*N/A*	*N/A*	*N/A*	F	72	6.17	ε4/ε4	NBB-IBB
No. 13	AD	V/VI	4-5	Frequent	*N/A*	M	62	1.75	ε3/ε4	NBB-IBB
No. 14	AD	V/VI	4-5	Moderate	*N/A*	M	73	10.25	ε2/ε4	NBB-IBB
No. 15	AD	VI	5	*N/A*	*N/A*	M	90	28.00	ε3/ε4	NeuroCEB
No. 16	DSD	VI	5	*N/A*	*N/A*	F	56	28.50	ε3/ε4	KCL
No. 17	DSD	V	*N/A*	*N/A*	*N/A*	F	64	8.00	ε3/ε3	Cambridge
No. 18	DSD	V	*N/A*	*N/A*	*N/A*	M	52	7.00	ε3/ε3	Cambridge
No. 19	DSD	VI	*N/A*	*N/A*	*N/A*	M	55	11.00	ε3/ε3	Cambridge
No. 20	DSD	V	*N/A*	*N/A*	*N/A*	M	67	3.00	ε3/ε3	Cambridge

Supplementary Table S1 I Demographics of the post-mortem human hippocampal tissues used in the study. Abbreviations: AD = Alzheimer's disease; DSD = Down syndrome dementia; PDD = Parkinson's disease dementia; ApoE = ApoE genotype; PMI = post-mortem interval (in hours); AD Braak = Braak tau stage; PD Braak = Braak α-synuclein stage; Thal phase = Thal phase for amyloid beta plaques; CERAD score = CERAD neuritic plaque score; M = male; F = female; N/A = not available; NBB-IBB = Neurobiobank of the Institute Born-Bunge; NeuroCEB = National Brain Bank Neuro-CEB; KCL = King's College London Brain Bank; Cambridge = Cambridge Brain Bank.

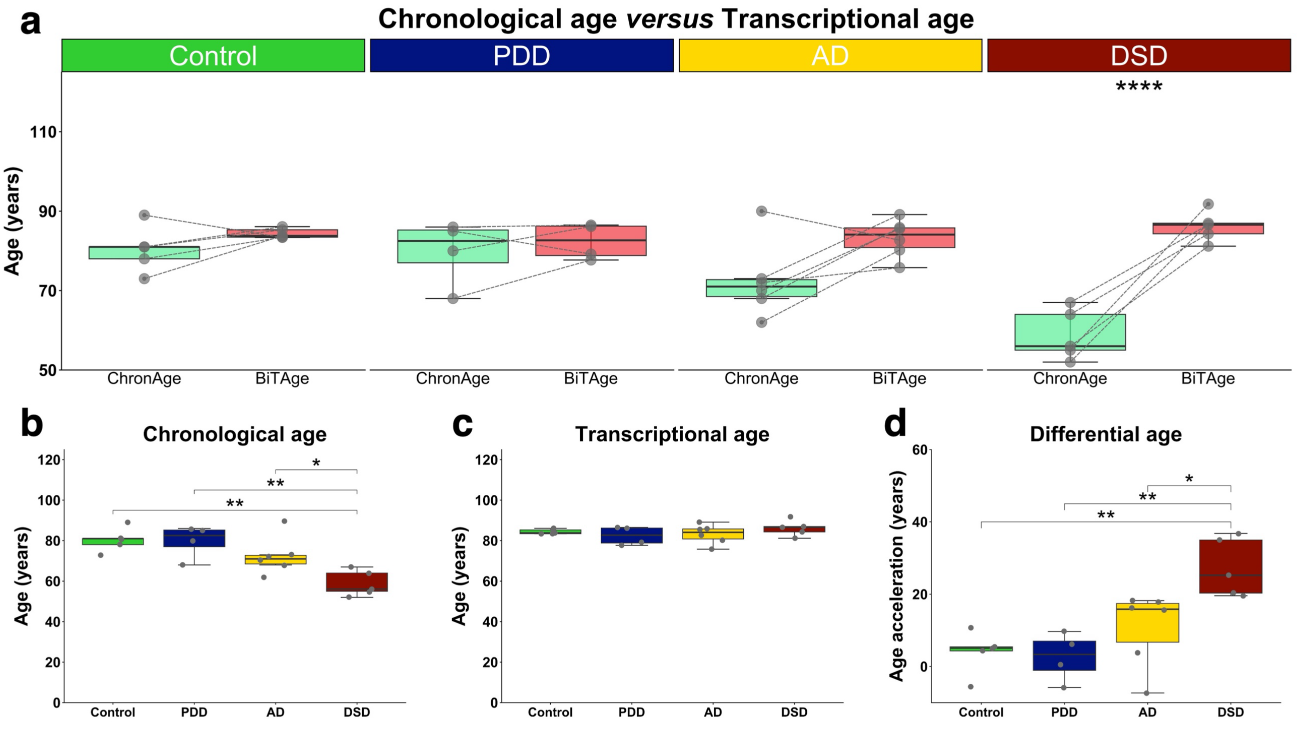

Supplementary Fig. S1 I Comparisons of the chronological age, transcriptional age, and differential age with the BiT age algorithm. **a** Transcriptional age (BiTAge) was calculated with the BiT age algorithm for each sample and compared against the chronological age (ChronAge) of the same individual, which are connected through a dotted line. Down syndrome demented (DSD) samples showed to have a higher transcriptional age than their biological age (Control, p-value = 0.965, PDD, p-value = 0.998; AD, p-value = 0.078; DSD, p-value = 0.000001). **b** Chronological age (or age at death) in years was 80.4 ± 5.8, 79.8 ± 8.3, 72.5 ± 9.4, and 58.8 ± 6.4 for the Control, PDD, AD, and DSD groups, respectively. The individuals with DSD were significantly younger at death compared to the other groups (Control *versus* DSD, p-value = 0.00209; PDD *versus* DSD, p-value = 0.00452; and AD *versus* DSD, p-value = 0.0428). **c** There was no difference in transcriptional age between the groups (84.4 ± 1.2, 82.4 ± 4.6, 83.2 ± 4.7, and 86.2 ± 3.9 for the Control, PDD, AD, and DSD groups, respectively). **d** Age acceleration was calculated by the subtraction of the chronological age from the transcriptional age, which was represented as the differential age. Individuals with DSD had a significantly acceleration in age compared to the other groups (Control *versus* DSD, p-value = 0.00184; PDD *versus* DSD, p-value = 0.00187; and AD *versus* DSD, p-value = 0.0189). After normal distribution was assessed with the Shapiro-Wilk test and the assumption for homoscedasticity controlled, statistical significance was tested using the two-way ANOVA or the one-way ANOVA followed by the Tukey's HSD *post hoc* test. Statistical significance was presented with * p-value ≤ 0.05; ** p-value ≤ 0.01; *** p-value ≤ 0.001.

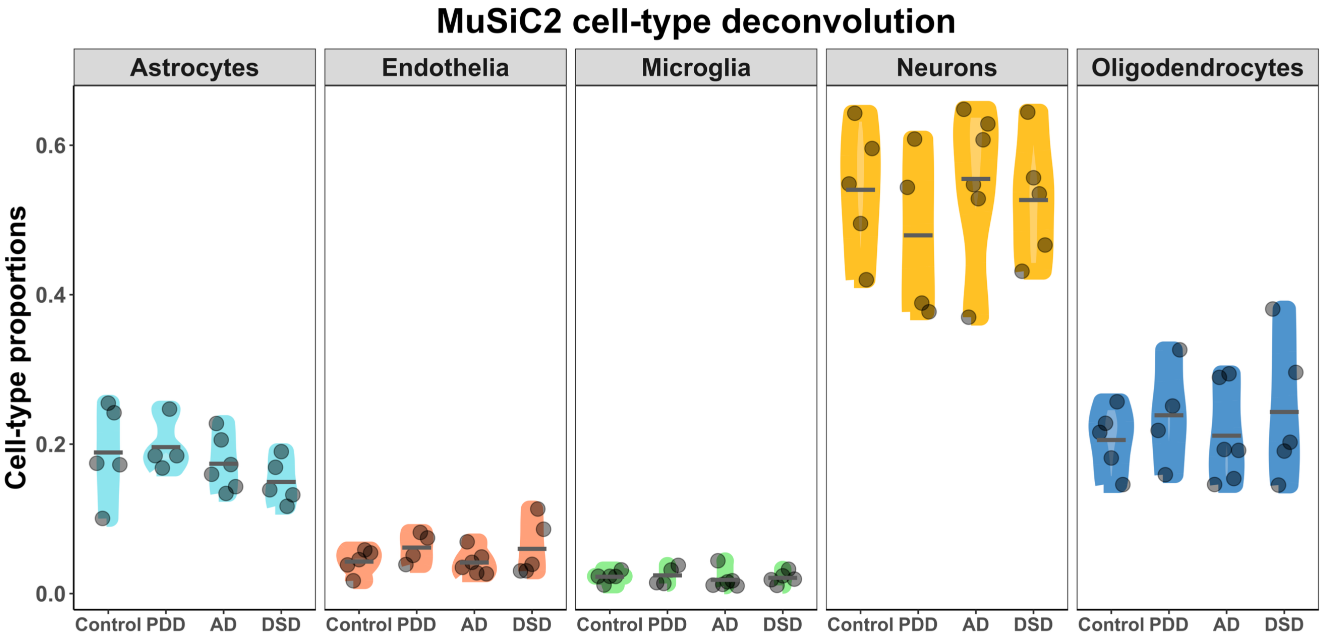

Supplementary Fig. S2 I Violin plot for astrocytes, endothelia, microglia, neurons and oligodendrocytes cell-type deconvolution. The cell-type composition was inferred with the MuSiC2 algorithm for the bulk RNA-sequencing data from non-demented (Control), Parkinson's disease with dementia (PDD), Alzheimer's disease (AD) and Down syndrome with dementia (DSD) cases. For each cell-type, the solid line represents the average proportions across samples, which was inferred through bulk data deconvolution with the single-cell RNA-sequencing dataset obtained from Darmanis *et al.*, 2015. No single cell-type significantly differed between groups. Statistical significance was tested using the one-way ANOVA followed by the Tukey's HSD *post hoc* test.

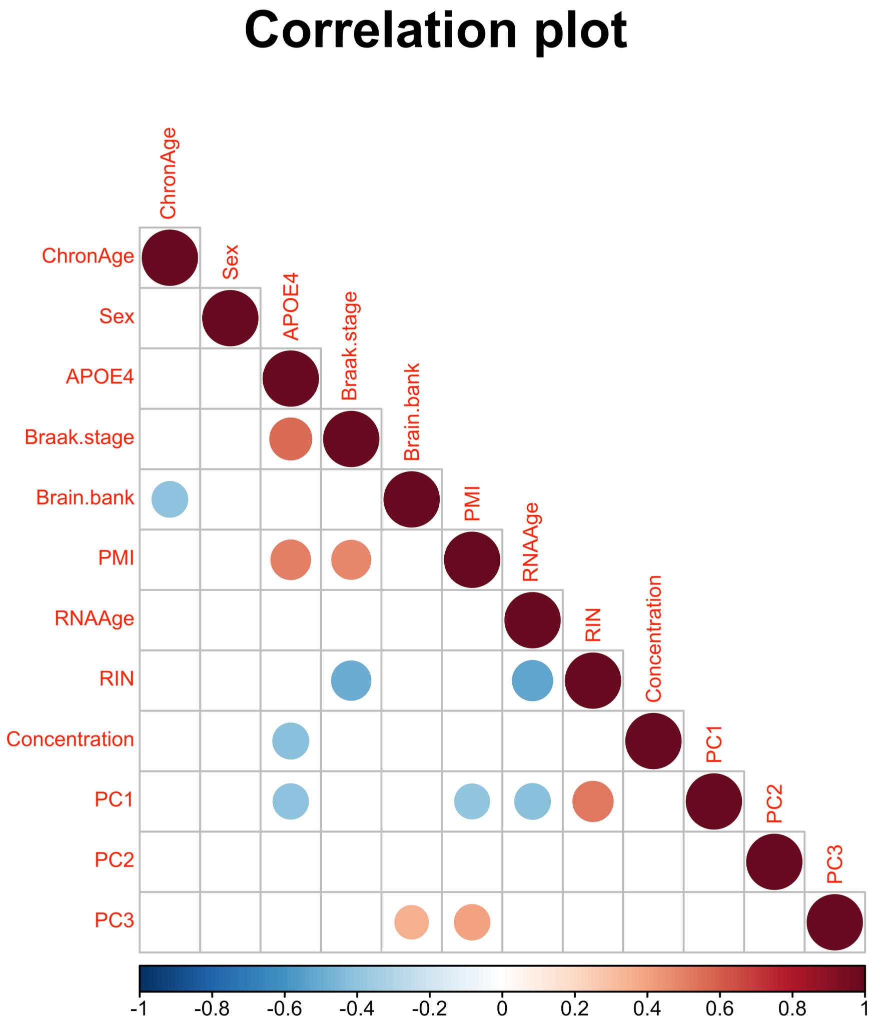

Supplementary Fig. S3 I Kendall's Tau correlation between potential sources of biological variation and the first three principal components.
Circle sizes are proportional to the correlation coefficient, while the colors indicate positive (red) and negative (blue) coefficients. Non-significant pairwise correlations (p-value > 0.05) are not presented in the plot.

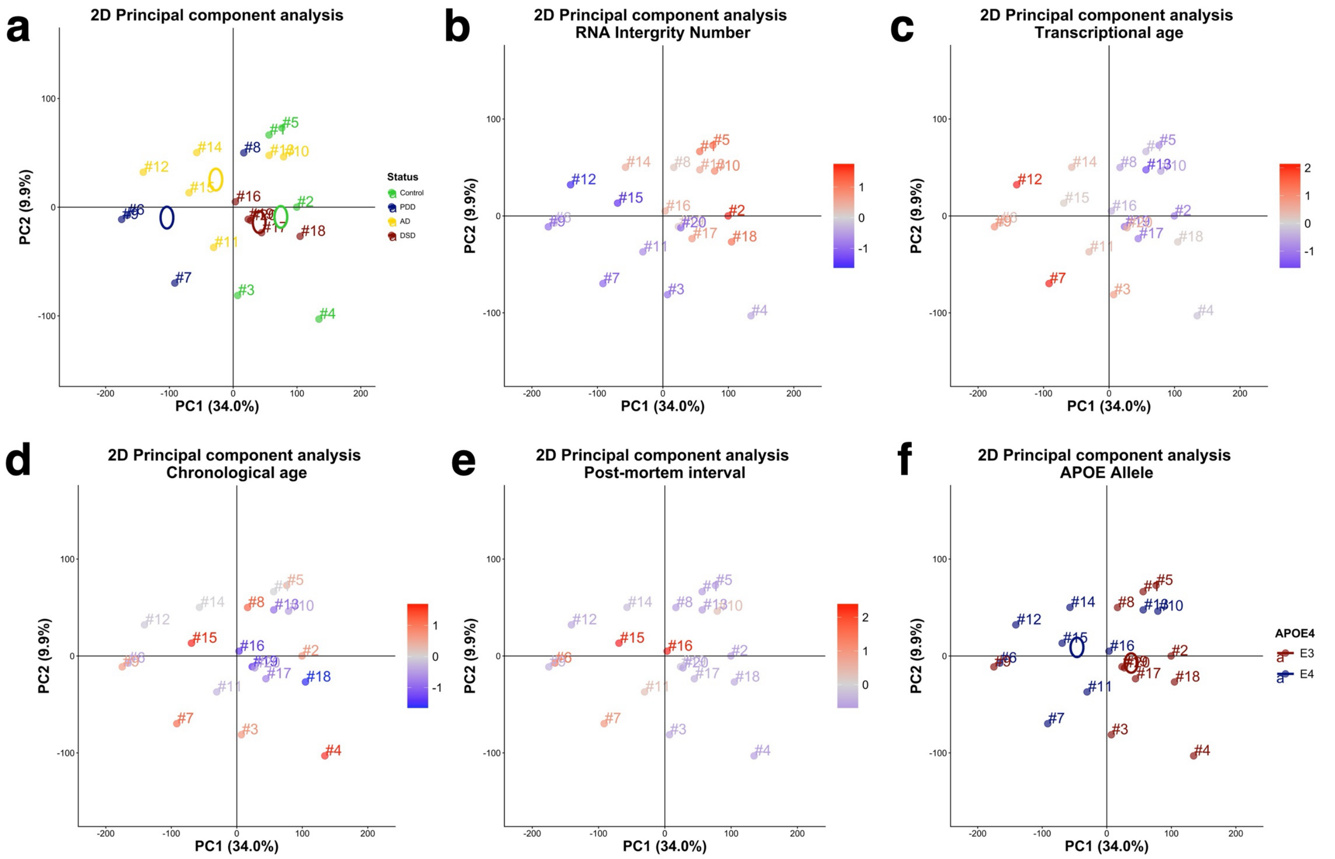

Supplementary Fig. S4 | Two-dimensional principal component analysis cluster plots of the RNA-sequencing dataset.
The distribution of **a** the sample groups by their status, **b** RNA integrity number, **c** transcriptional age (RNAAge), **d** chronological age, **e** post-mortem interval, and **f** APOE status.

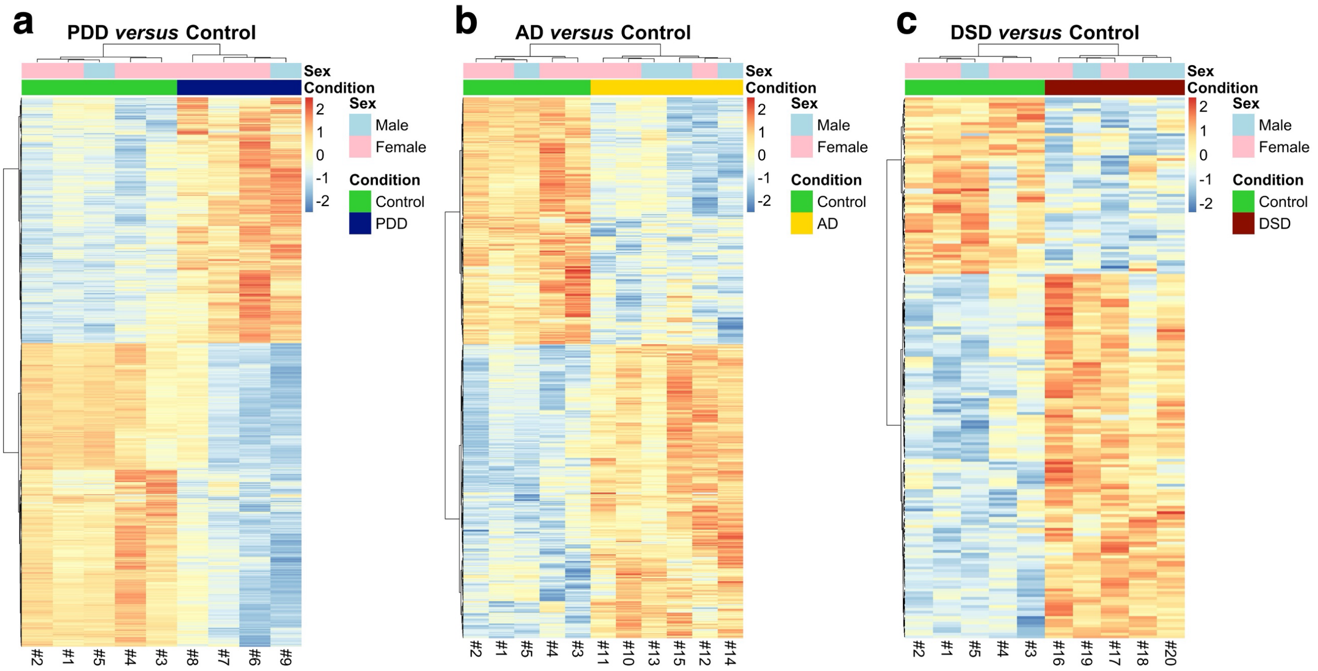

Supplementary Fig. S5 | Heatmaps of differentially expressed genes (DEGs).
a Heatmap showing the DEGs between Parkinson's disease dementia (PDD) and non-demented cases (Control). **b** Heatmap showing the DEGs between Alzheimer's disease (AD) and Control. **c** Heatmap showing the DEGs between Down syndrome dementia (DSD) and Control. Red lines indicate upregulated genes, whereas blue lines represent downregulated genes.

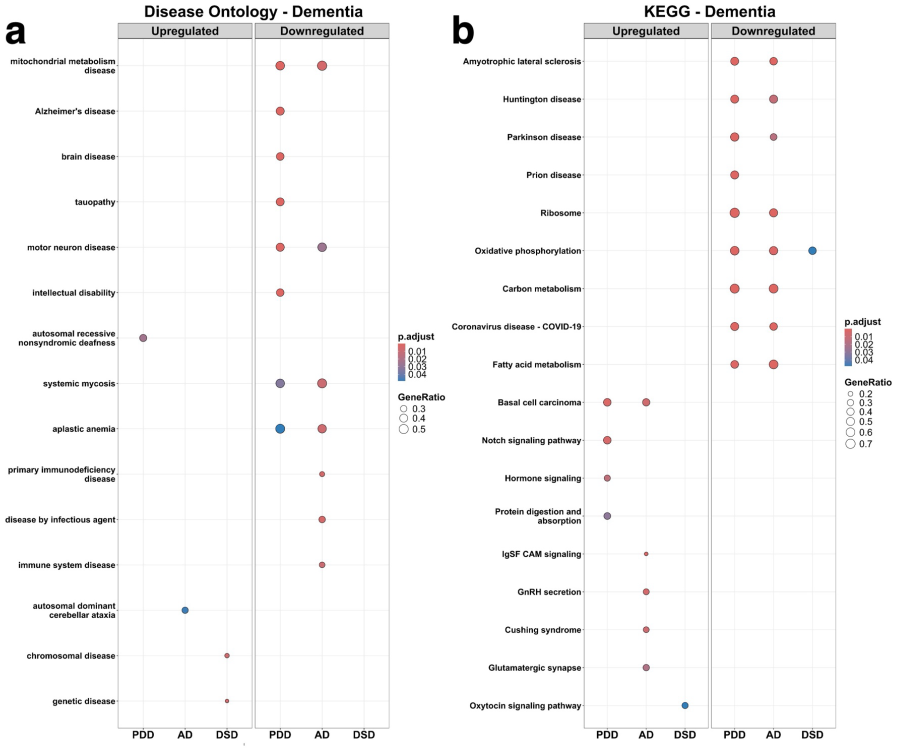

Supplementary Fig. S6 | Disease Ontology (DO) and Kyoto Encyclopedia of Genes and Genomes (KEGG) analysis for the samples of Parkinson's disease dementia (PDD), Alzheimer's disease (AD) and Down syndrome dementia (DSD).
a DO terms that were significantly enriched for PDD, AD and DSD individuals. **b** KEGG terms that were significantly enriched for PDD, AD and DSD individuals. In each plot, dot size indicates the number of genes in that gene set by ratio (GeneRatio) and dot color reflects statistical significance based on FDR-adjusted p-values (p.adjust).

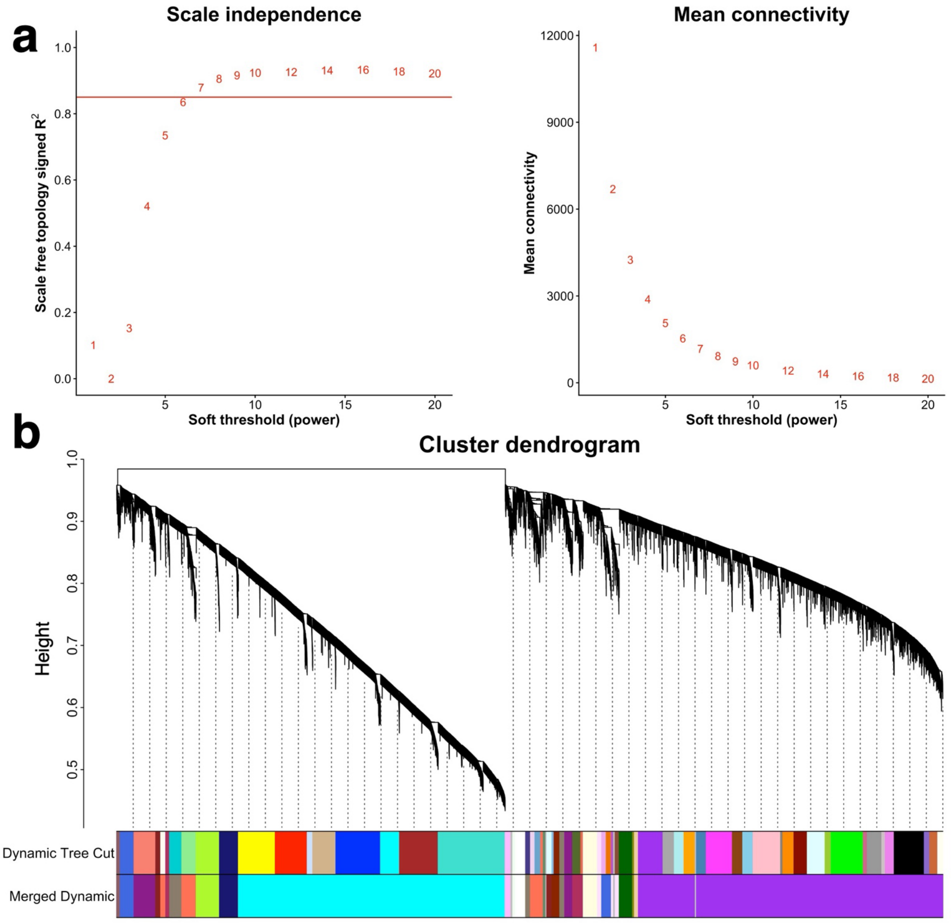

Supplementary Fig. S7 | Weighted gene co-expression network analysis (WGCNA).
a Relationship between various soft-thresholding powers (β) and scale-free topology fit indices (left). Relationship between mean connectivity and various soft-thresholding powers (right). **b** Hierarchical clustering dendrogram of genes, with modules represented by different colors. Genes with the highest median absolute deviation enriched modules in a co-expression network (Dynamic Tree Cut). After merging high related modules (cutoff value ≤ 0.25), a total of 22 co-expression clusters were identified (Merged Dynamic).

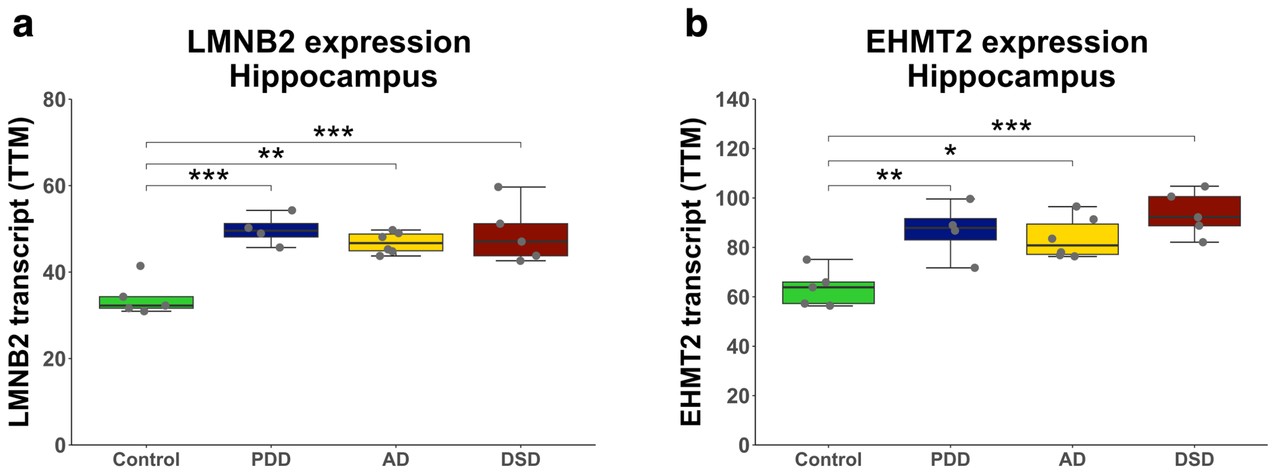

Supplementary Fig. S8 I Hub gene expression in the normalized count matrix with the Trimmed Mean of M-values (TMM) method.
a Normalized LMNB2 expression levels in the hippocampus obtained from the count matrix for the samples of non-demented controls (Control), Parkinson's disease dementia (PDD), Alzheimer's disease (AD) and Down syndrome dementia (DSD). **b** Normalized EHMT2 expression levels in the hippocampus obtained from the count matrix for Control, PDD, AD and DSD samples. Statistical significance was tested using the one-way ANOVA followed by the Tukey's HSD *post hoc* test, which was presented with * p-value ≤ 0.05; ** p-value ≤ 0.01; *** p-value ≤ 0.001.
